# Computational modeling of plant root development: the art and the science

**DOI:** 10.1111/nph.70164

**Published:** 2025-04-23

**Authors:** Kirsten H. ten Tusscher

**Affiliations:** ^1^ Experimental and Computational Plant Development, IEB, Department of Biology Utrecht University Winthontlaan 30C 3526 KV Utrecht the Netherlands; ^2^ Theoretical Biology, IBB, Department of Biology Utrecht University Winthontlaan 30C 3526 KV Utrecht the Netherlands

**Keywords:** cell types as attractors, clock‐and‐wavefront, computational modeling, morphogen gradient, network motifs, plant root development, reflux‐and‐growth, Turing patterns

## Abstract

Plant root development, like any developmental process, arises from the interplay between processes like gene expression, cell–cell signaling, cell growth and division, and tissue mechanics, which unfold over a wide range of temporal and spatial scales. Computational models are uniquely suited to integrate these different processes and spatio‐temporal scales to investigate how their interplay determines developmental outcomes and have become part of mainstream plant developmental research. Still, for non‐modeling experts, it often remains unclear how models are built, why a particular modeling approach was chosen, and how to interpret and value model outcomes. This review attempts to explain the science behind the art of model building, illustrating the simplifications that are often made to keep models simple to understand and when these are and are not justified. Similarly, it discusses when it is safe to ignore certain processes like growth or tissue mechanics and when it is not. Additionally, this review discusses a range of major breakthrough modeling articles. Their approaches are linked to classical concepts and models in developmental biology like the French flag positional information gradient of Lewis Wolpert and the repetitive patterning mechanism proposed by Turing, in addition to highlighting the lessons they taught us on plant root development.

**Table:** 

	Contents	
	Summary	2446
I.	Introduction	2446
II.	Results	2447
III.	Discussion	2458
	Acknowledgements	2459
	References	2459

## I. Introduction

Plant development is an inherently complex process, involving processes ranging from the millisecond‐to‐minute range such as receptor‐ligand signaling and auxin transport, to the minute‐to‐hour scale responses in gene expression regulation, cell growth, division and differentiation dynamics, the hour‐to‐day consequences in tissue‐scale zonation, cell fate and mechanics patterning, and the day‐to‐week effects on shoot or root system architectures this has. Furthermore, these processes are not arranged on a linear scale where the smaller spatial and temporal scale processes drive the larger scale ones (Noble, 2008). Instead, regulatory interactions occur between all levels, with, for example, tissue‐level mechanical stresses and strains feeding back on signaling and transport patterns (Heisler *et al*., 2010), or cell division behavior shaping morphogen gradients (Mähönen *et al*., 2014). In addition to the complexities involved in animal development, further complexity in plants arises from the extensive interplay between developmental processes and environmental conditions to enable plastic adaptation of plant morphology to its environment.

Because of these complexities, it has long been recognized that mathematical and computational modeling, through integrating the various spatial and temporal scales at which relevant processes occur as well as their feedback interactions, is a valuable tool in enhancing our understanding of plant developmental processes. By now classical is the work of Alan Turing, who demonstrated mathematically how regular spatial patterns in biology could arise from the combination of a short‐range positive feedback and a longer range negative feedback regulation. While his work was of a highly abstract nature, it was strongly inspired by processes like plant phyllotaxis (Turing, 1952). Also famous within the plant field is the work of Tsvi Sachs, who postulated that a positive feedback between the flux of auxin in a certain direction and the strength of that flux could drive the canalization of auxin flux into vascular patterns (Sachs, 1969, 1981). Later research building on these classics has shown the critical role of the PIN membrane proteins responsible for directed auxin transport in both phyllotaxis and venation (Feugier & Iwasa, 2006; Fujita & Mochizuki, 2006; Smith *et al*., 2006; Heisler *et al*., 2010; Bhatia *et al*., 2016), while also highlighting additional complexities (Heisler *et al*., 2010; Besnard *et al*., 2014a,b; Ravichandran *et al*., 2020).

Mathematical and computational models have become increasingly integrated into mainstream plant developmental biology. Still, it often remains elusive why models were constructed in a particular way, or how to value the quality of a model. Indeed often, model construction is described as partly art or craft and partly science, further adding to the seeming magic performed by models. In this review, I will attempt to, at least partly, demystify the ‘how’ and ‘why’ of mechanistic model building for non‐modeling experts, while trying to inspire fellow modelers to keep these aspects in mind when describing their approaches. I will focus this discussion on work related to plant root development and will relate the discussed modeling studies to classical concepts and models from both plant and animal development.

## II. Results

### 1. How are good models built?

#### Mechanistic models

To answer this question, one must first consider what makes a model a good model, and for that, I need to discuss how this depends on the purpose that a model may serve. If one thinks, for example, of a miniature car or train, or the map of the London metro net, the purpose of those models is merely to represent a detailed yet idealized representation of reality. Such models should be considered as descriptive and aid little in either generating predictions or providing understanding. If instead one thinks of weather, climate, or stock market models, the main purpose of the model is to predict the future in as much quantitative detail as possible. These are thus predictive models yet offer due to their complexity limited insight. The same holds true for the increasingly popular models generated using machine learning, a branch of artificial intelligence using algorithms to learn patterns from data. Due to their so‐called black box nature, that is, the non‐transparent relation between inputs and outputs, these again excel in quantitative predictiveness yet offer limited insight. By contrast, the type of models typically being developed in the field of plant developmental biology serve an entirely different purpose, namely to obtain an understanding of the mechanisms driving the biological process of interest. These models are often described as mechanistic models.

**Fig. 1:**
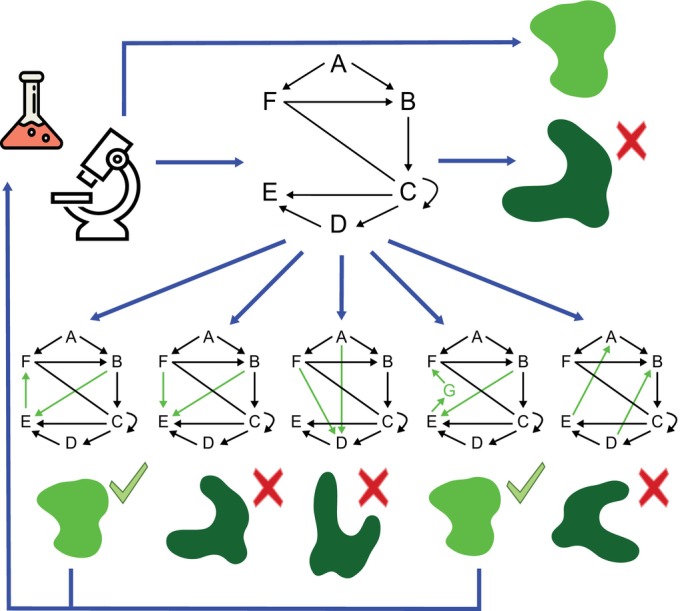
Use of models to identify most likely missing players and interactions. Experimental data provides us with knowledge of the involved players and interactions (big network) as well as the eventual phenotypic output of the biological process of interest (bright green blob). Often, a model incorporating the network will however fail at reproducing this phenotypic output (dark green blob). The model can then be used to try a large number of additional interactions and players (smaller networks, bright green arrows and letters) to investigate which of these changes would result in the correct phenotypic output. Only the subset of interactions and players found to produce the correct output then need to be tested experimentally to investigate which of these hypothetical mechanisms occurs *in planta*. Blue arrows indicate the flow of the research process combining experiments and modeling. Black arrows indicate the regulatory interactions discovered in the first round of experimentation.

In contrast to what one may naively assume, a good mechanistic model should not incorporate as much of the known details – genes, peptides, hormones, signaling cascades, cell types, tissue mechanics, etc. – as possible. Increased complexity comes at the cost of, not only increased computational demands and a larger number of model parameters for which values should be found, but also reduced ease of understanding. Instead, models should, at least initially, be kept as simple as possible. Given that a model's outcome can logically only depend on the players incorporated in a model, this enables one to determine whether the currently incorporated players and their interactions suffice to reproduce the phenomenon of interest and to pinpoint exactly how this works. Furthermore, relatively simple models allow for a large number of efficient simulations, enabling us to try out which additional players or interactions would be the most likely candidate key factor or link missing from our current understanding (Fig. 1). As such, these models help us direct our experimental efforts to where these are expected to be most informative. Indeed, for modeling to substantially contribute to our understanding, an intermediate level of knowledge is optimal, with little knowledge hampering model construction and full knowledge making it obsolete.

#### The right level of detail

**Fig. 2:**
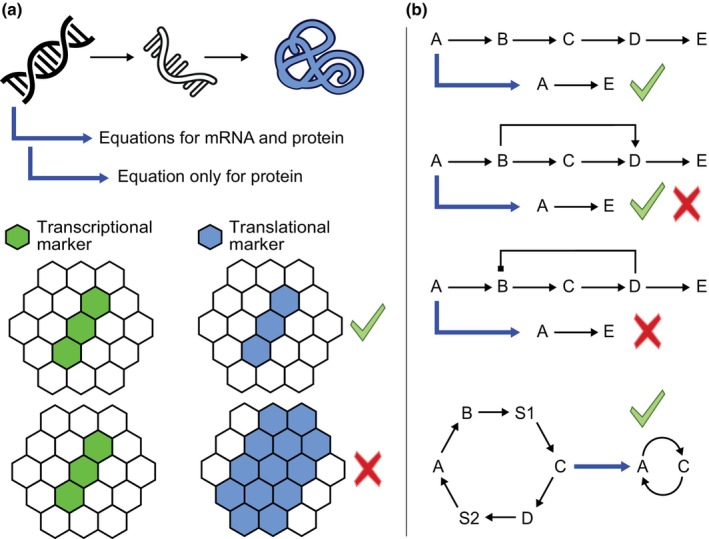
Frequently used model simplifications and when these can be made safely. (a) In many models, instead of using separate equations for mRNA and protein, describing transcription, translation, and both mRNA and protein turnover, a single equation for only protein dynamics is used. If the transcription and translation domains are identical, and if regulation can be captured in a single equation, this simplification is fine. If, for example, a protein is far more stable than the mRNA and/or is mobile from cell to cell, the translational domain expands beyond the transcriptional domain, and this simplification should not be made. (b) Linear chains of causation in gene expression regulation or signaling can often be simplified, allowing a reduction in the number of model variables. However, in the case of feedforward regulation, this simplification may work for some but not all conditions. For example, the feedforward motif may offer redundancy (simplification is ok) or function in filtering noise (simplification is not ok). In the case of negative feedback regulation, a simplification ignoring this feature will always miss major functional aspects, while on the other hand, a long positive feedback cycle may be summarized by a shorter one if temporal delays are not important. Black arrows indicate regulatory interactions, and blue arrows indicate how from the original system a more simplified version is derived to keep models simple.

The above begs the question of how one determines what should or should not be incorporated in a model. While this process may be seen by some as an art or craftsmanship rather than following clearly laid out rules, there is neither mystery nor magic to it. Model building requires considerable knowledge of the developmental process of interest, to know what the key genes, hormones, interactions, cellular behaviors, mechanical processes, etc. are taking part in the developmental phenomenon that should be at least considered for inclusion. Additionally, it requires knowledge of the interactions between players and processes as well as relevant temporal and spatial scales to recognize which of these players and interactions will likely be needed to answer the particular research question. As an example, it may often not be necessary to separately model mRNA and protein levels; therefore, modelers often use a single equation for the gene product, collapsing transcription and translation into a single process and ignoring mRNA degradation (see, e.g. Cruz‐Ramírez *et al*., 2012) (Fig. 2a). However, if mRNA and protein expression domains significantly differ due to, for example, prolonged protein stability and movement of the protein outside of the transcription domain (Mähönen *et al*., 2014), this distinction does become relevant (Fig. 2a). Similarly, if a gene A activates an important developmental gene E through intermediary genes B, C, and D, one may want the model to simplify and let gene A directly control gene E, thereby saving three model variables and even more model parameters. This simplification is fine if no further feedback regulations (e.g. C negatively affecting the upstream B) affecting the overall dynamics occur, and if temporal delays because of the relay of genes are small. Similar arguments apply for signaling instead of gene regulatory interactions (see, e.g. el‐Showk *et al*., 2015). Thus, if in between steps are expected to be inconsequential, these can be ignored to keep models simple, while if feedbacks, delays, or other complexities are suspected, it is better to incorporate these and evaluate their consequences (Fig. 2b). In this respect, it is important to realize that model building, as much as doing follow‐up experiments, is an iterative process. Puzzle pieces are added as long as the model is not in agreement with experimental data, yet puzzle pieces not (significantly) contributing may also be removed again. While modelers generally strive for simplicity, models should not be too simple. More specifically, if a model, for example, strives to explain a complex spatiotemporal pattern in protein X, the model should include gene X but not prepattern when and where gene X is expressed (Clark *et al*., 2020; Perianez‐Rodriguez *et al*., 2021). Instead, the model should allow the pattern of X to dynamically unfold from the processes included that impinge on X (Fig. 2b).

#### Model robustness vs hypothesis discrimination

In addition to including the right level of detail, a good mechanistic model should be robust; for example, the desired behavior should not depend on ultra finely tuned parameter values. Critical dependence on parameter values would imply that model behavior is not a generic result of the processes and interactions incorporated, but rather a highly rare model behavior that should be mistrusted. This is why modelers are asked to perform a sensitivity/robustness analysis to demonstrate that qualitatively similar behavior arises for moderate changes in parameter values. Also, if a model includes unknowns, these hypothesized factors or interactions should at least be plausible and provide testable predictions. Of course, models should display different behaviors when, for example, simulating wild‐type vs mutant or a control vs hormone treatment. Additionally, a model should ideally display different behaviors when simulating different hypotheses for a certain process; otherwise, these cannot be discerned through experimental validation.

While the above deals with the level of detail at which processes are modeled, the required robustness for assumed parameter values and the capacity to tell apart hypotheses, another question is which types of processes to include in a model, how to include the spatial nature of developmental processes, or what level of quantitative detail is needed. As a concrete example, often gene expression and hormone signaling are described using the so‐called differential equations, describing the rate of changes in these factors and allowing these factors to take on continuous values. However, this comes at the cost of requiring parameter values for production, degradation, and affinity for all factors involved. Put simply, the more parameters a model has, the more data are required to determine parameter values by fitting the model to the data. Therefore, in cases where one wants to describe a large number of genes and where it is mostly relevant whether genes are highly expressed or not and not so much their precise value or how long it took for them to reach that value, instead a so‐called Boolean approach is used. In this alternative modeling approach, genes can be either on (expressed) or off (not‐expressed) and this depends on the state of the genes regulating them following simple rules. Similarly, while natural developmental processes typically involve cell growth, division, and elongation, as well as tissue mechanics, many models exist in which growth or mechanics are not incorporated. Here as well, rationally informed choices are made, with, for example, only modeling root asymmetric auxin patterning under tropic responses (Band *et al*., 2012; van den Berg *et al*., 2016; Retzer *et al*., 2019) while ignoring root bending based on the fact that it is the auxin asymmetry that is driving the asymmetric growth and assuming feedback effects of bending on auxin patterning can be ignored (but see Laskowski *et al*., 2008, where root bending and the cell shape changes it induces do affect auxin levels). On a similar note, often the patterning upstream of the cell fate, cell division orientation, and zonation decisions are modeled, ignoring the downstream division and growth patterns (Cruz‐Ramírez *et al*., 2012; el‐Showk *et al*., 2015; García‐Gómez *et al*., 2017). This approach is generally acceptable unless the stability of components results in spread beyond their transcriptional domain (Mähönen *et al*., 2014) or if you are interested in addressing how growth mechanics may feedback on the patterning (Marconi *et al*., 2021; Ötvös *et al*., 2021).

In the next sections, I attempt to clarify how modeling choices are made by relating concrete computational plant development models to classical models and concepts.

### 2. Zonation

**Fig. 3:**
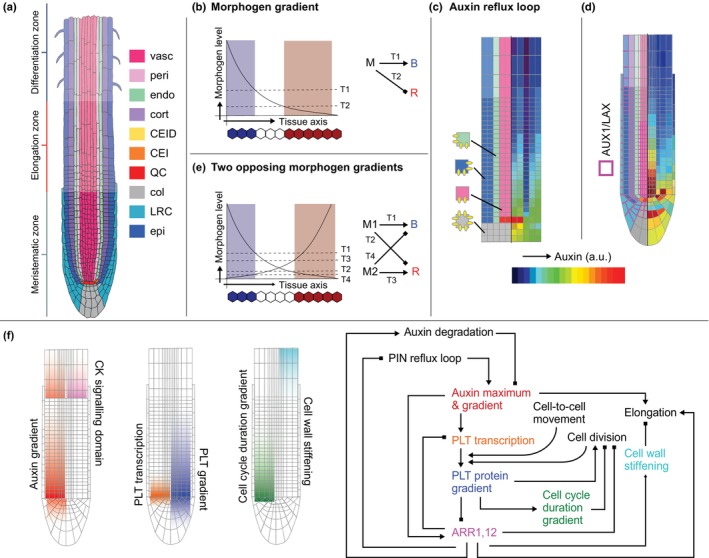
Zones and their patterning in the Arabidopsis root tip. (a) Arabidopsis root tip anatomy with the different cell types and developmental zones. Epi, epidermis; cort, cortex; endo, endodermis; peri, pericycle; vasc, vasculature; QC, quiescent center; CEI, cortex endodermis initial; CEID, cortex endodermis initial daughter cell; LRC, lateral root cap. Root illustration modified from the Plant Illustration repository (2017). (b) Illustration of the classical French flag model proposed by Lewis Wolpert. A gradient of the morphogen (M, black line), through exceeding different threshold levels (T1, T2, horizontal dashed lines) at different locations can result in the positional specification of different cell types (blue, white, and red). A simple way to operationalize this is by having the morphogen induce (lines with arrows) the ‘blue’ gene when M > T1, and the morphogen repressing (lines with blunt ends) the ‘red’ gene when M > T2. (c) Illustration of the PINF‐FORMED (PIN) efflux patterning and resulting auxin pattern in the Grieneisen *et al*. (2007) model. Note how a reasonably realistic auxin gradient was generated while no realistic root tip architecture, and no further details on auxin transport and metabolism and modulation by other players were included. (d) Illustration of how adding the active AUXIN1(AUX1) and LIKE AUX1 (LAX)‐mediated auxin import and a realistic root tip geometry incorporated in the Band *et al*. (2014) paper resulted in a refined model auxin pattern. (e) Illustration of positional information‐based patterning in the presence of two opposing morphogen gradients (M1 and M2) that have opposing effects on the ‘blue’ and ‘red’ genes. (f) Illustration of how a succession of models from various groups have brought our insights into root zonation further. Left are modeled hormone and growth patterns; right is the regulatory network when integrating these studies, with matching colors for the various regulators to indicate their patterns (left) and place in the regulatory network (right). Integrating the high auxin levels‐driven expression of PLETHORA (PLT) and the stable PLT protein model enabled cell‐to‐cell movement and cell division inheritance in the Mähönen *et al*. (2014) model, which explains experimentally observed PLT transcriptional (orange) and translational (blue) domains. Superimposing elongation zone‐centered cytokinin signaling (purple) antagonizing auxin transport and levels in the Di Mambro *et al*. (2017) paper predicted an auxin minimum at the transition zone boundary and a secondary rise in auxin in the elongation zone (red). Incorporating auxin induction of cytokinin signaling AUXIN RESPONSE REGULATORS (ARRs) and a mutual repression between PLTs and cytokinin signaling, Salvi *et al*. (2020) next explained the EZ‐centered domain of cytokinin signaling and how it is set up during post‐embryonic root development. In subsequent studies, Echevarria *et al*. (2022) demonstrated how the PLT gradient translates into a cell cycle duration gradient (green), while Liu *et al*. (0096) focused on the mechanics of tissue growth to elucidate how the cytokinin (CK) domain triggers the cell wall stiffening that terminates elongation (cyan).

In plants, because of the rigid cell walls that cells share with their neighbors, cells cannot change their relative position. Consequently, all plant growth is fueled by localized stem cell niches. In plant roots, these niches consist of a quiescent center (QC) with infrequently dividing cells, surrounded by more frequently dividing daughter cells that provide the initial cells for the different tissue layers. The stem cell niche thus delivers cells into the meristem in which cells undergo rapid transit‐amplifying divisions, after which they enter the elongation zone to undergo vacuolar expansion, and finally enter the differentiation zone in which cells attain their final differentiated cell fate (Fig. 3a). Thus, an important question is how cells know in which zone they reside and hence which developmental behavior to execute.

#### Morphogen gradients

In the late 1960s, Lewis Wolpert (1968, 1969) proposed his so‐called French flag model. He proposed that a gradient of an informative signal, called a morphogen, would enable cells at different positions to execute a different program and thereby attaining a different fate (Fig. 3b). While the root cells do not attain a different final fate at the distinct zones, but rather traverse these zones and sequentially execute different developmental behaviors (Rutten & Ten Tusscher, 2019), still a positional information gradient would be a logical candidate to enable plant cells to achieve this. Indeed, Sabatini *et al*. (1999) demonstrated the existence of an auxin gradient in the root tip, with a maximum in the QC and auxin levels tapering off through the meristem that was shown to be important for root zonation, as further confirmed by a later study (Petersson *et al*., 2009). While it has long remained debated whether auxin could be considered a true morphogen, a concrete question to address was the mechanism giving rise to this auxin gradient.

In a seminal paper, Grieneisen *et al*. (2007) simplified root tip architecture to a simple rectangular shape, focusing on the role of PINF‐FORMED (PIN) mediated polar auxin export in auxin patterning. The authors simulated both naturally observed PIN patterns and alternative patterns not observed in nature and not easily created through mutations. This enabled them to demonstrate how the experimentally observed reflux‐loop, with downward‐oriented PINs in the vasculature, apolar PINs in the columella, and upward and inward PIN orientations in the ground tissues (Blilou *et al*., 2005) is essential for generating a robust auxin gradient (Fig. 3c). In a follow‐up study, Grieneisen *et al*. (2012) made further use of the unique capacity of models to try out alternatives and compare these to the naturally occurring mechanism. Specifically, the authors compared the PIN reflux loop with classical local production–global decay and directional transport mechanisms for gradient formation. This enabled them to demonstrate that given the rapid diffusion of auxin and its relatively long lifespan, only a reflux‐loop mechanism enables gradient formation within a reasonable time frame. Additionally, through repatterning PIN polarities, the mechanism enables a rapid adjustment of auxin patterns in response to tropic stimuli (Kleine‐Vehn *et al*., 2010) or when forming new lateral roots (Benková *et al*., 2003). Through incrementally adding details to these types of models, the Band laboratory showed the importance of detailed root tip architecture, active AUXIN1 (AUX1) and LIKE AUX1 (LAX)‐mediated auxin import (Fig. 3d), auxin metabolism, cooperation between PIN and ATP‐BINDING CASSETTE (ABCB) carrier‐mediated auxin export, and passive plasmodesmatal auxin transport in fine‐tuning auxin patterns (Band *et al*., 2014; Mellor *et al*., 2016, 2020, 2022). Additionally, the work of the Lindsey laboratory demonstrated the important interplay between auxin, ethylene, and cytokinin signaling in further refining the auxin pattern (Moore *et al*., 2015, 2017).

In 2014, Mahonen *et al*. demonstrated that while auxin levels determine rates of cell division, elongation, and differentiation, it is PLETHORA (PLT) protein levels that determine whether a cell acts as a stem cell, transit‐amplifying cell, or starts elongation and differentiation. This implied that PLTs, rather than auxin, act as a zonation morphogen. Surprisingly, their experiments also indicated that, while auxin induces PLT transcription and PLT protein gradients resemble the auxin gradient, the PLT transcriptional domain is confined to the stem cell niche. Thus, how then is the PLT protein gradient generated? Adding equations for auxin‐mediated PLT expression, protein production, cell‐to‐cell movement, and turnover to a root tip auxin transport model, Mahonen *et al*. first demonstrated that to reproduce the observed narrow transcriptional domain, PLT transcription must require high auxin levels. Next, the model predicted that to translate this narrow transcriptional domain into an extended protein domain, PLT proteins must be long‐lived. Using the model, the authors demonstrated that this protein stability enabled both slow plasmodesmata‐mediated cell‐to‐cell movement and cell lineage transport, the inheritance and subsequent slow degradation‐mediated dilution of PLT protein in cells that, through growth and division, are pushed out of the transcriptional domain (Ibañes *et al*., 2006) (Fig. 3f). Notably, for these findings, the incorporation of growth processes into the model was essential. In another study, Echevarria *et al*. (2022) observed that while PLTs were thus known to promote cell division, via inducing KRP5, PLTs also prolong the duration of the G1 cell cycle phase, suggesting a so‐called INCOHERENT FEED FORWARD LOOP (IFFL) type regulation of cell division. Modeling this IFFL in a root tip model with a superimposed PLT protein gradient demonstrated how this IFFL allows for translating only robust fold increases in PLT expression into substantial increases in cell cycle duration, consistent with experimental data showing only significantly prolonged cell cycle durations near the QC (Fig. 3f).

In developmental patterning, robustness and scaling are of major importance. Therefore, in many systems, opposing morphogen gradients are involved (Fig. 3e), famous examples being the Bicoid and Caudal gradients and the fibroblast growth factor (FGF)/wingless‐related integration site (WNT) and Retinoic Acid gradients involved in Drosophila and vertebrate anterior–posterior patterning (Dubrulle & Pourquié, 2004; Aulehla & Pourquié, 2010; Briscoe & Small, 2015). In plant roots, auxin enhances and cytokinin (CK) reduces the size of the meristematic zone (Dello Ioio *et al*., 2007, 2008). To investigate how cytokinin controls meristem size, Di Mambro *et al*. (2017) superimposed elongation zone localized CK signaling in a static root tip auxin transport model. The authors demonstrated that the experimentally observed CK‐mediated inhibition of PINs and the induction of auxin degradation results in an auxin minimum delimiting the meristem boundary and a secondary auxin rise in the early elongation zone (Fig. 3f). However, a question remained that what constrained AUXIN RESPONSE REGULATOR (ARR)‐mediated CK signaling to the elongation zone. Additionally, how do PLTs – the other positive regulators of meristem fate – fit in this picture? To answer these questions, in my laboratory, we extended our auxin‐PLT root growth model to include CK signaling while not superimposing its domain of action. The model predicted that, to explain ARR spatial patterning, an IFFL must be operating in which auxin‐induced ARR expression while via the PLTs also inhibiting ARRs (Salvi *et al*., 2020; Rutten & Ten Tusscher, 2021) (Fig. 3f). This IFF enables cells, as they grow, to increase their distance to the QC and PLT levels drop to start elongating and expressing ARRs. The secondary increase in auxin levels in the early EZ helps them to further fortify their ARR expression, with the ARR antagonism of both auxin and PLT further cementing the meristem boundary. These findings indicate that rather than a monotonically declining auxin or PLT morphogen gradient, high auxin + PLT levels control stemness (high levels) and divisions (lower levels), while the auxin + CK domain controls elongation.

Importantly, the above models assumed and thus superimposed a PIN reflux pattern and investigated what happens downstream, leaving open the question of how this reflux loop is formed. Mironova *et al*. (2012) demonstrated that incorporating different auxin optima for different PIN types enables the sequential patterning of the reverse fountain, yet required prepatterning of the different PIN expression domains and polarity patterns. More recently, Marconi *et al*. (2021) demonstrated how feedback between mechanics, auxin, and PIN polarization could drive reflux‐loop patterning. However, here prepatterned auxin influx in the vasculature and efflux from outer tissues was needed. An open question remains whether, perhaps combined, these mechanisms could drive truly self‐organized reflux loop patterning.

With the mechanisms behind meristem–elongation zone boundary patterning clarified, the next logical question is what controls the elongation zone–differentiation zone boundary? Given the importance of cell wall mechanics for cell elongation, Liu *et al.* (2022) constructed a 3D model for root growth mechanics. In this model, rather than hormonal‐genetic networks directly controlling cell growth, these networks instead impinge on cell wall stiffness. The interplay between wall stiffness and turgor pressure will subsequently result in cell growth and expansion. Using this approach, the authors were able to demonstrate the importance of CK‐mediated cell wall stiffening in the cessation of cell elongation, indicating CK involvement in both the start and termination of cell elongation (Fig. 3f).

### 3. Cell types and formative cell divisions

In addition to consisting of zones, roots consist of different cell types largely arranged in concentric cell layers, with the columella and lateral root cap at the outside covering only the meristematic zone, followed by the epidermis, cortex, endodermis, pericycle, and, in the middle, the vasculature (Fig. 3a). With all cells sharing the same DNA, differences between cell types need to arise from expressing different subsets of genes from this DNA.

#### Cell fates as alternative attractors

In the late 1960s, Kauffman (1969), using abstract models of gene regulatory networks, introduced the concept of different attractors (steady states of gene expression levels) as corresponding to different cell fates. Of course, gene expression regulation is only one layer of regulation (others being, e.g. DNA accessibility or protein stability), and besides, genes also hormonal, peptide, and RNA signals play key roles, yet the concept can be straightforwardly extended to multilayer regulatory networks. The attractor concept has become highly influential in animal and plant developmental biology as well as cancer research (Kauffman, 1971; Huang & Ingber, 2006). It enabled our understanding of how cells, by starting from different initial conditions, resulting from either noise or different positional inputs, can converge on a different final set of genes being expressed.

**Fig. 4:**
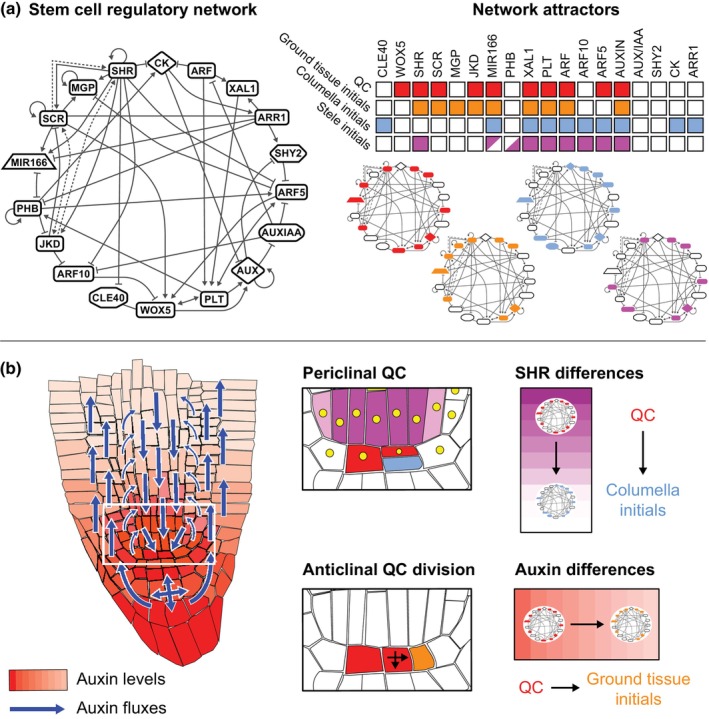
Cell types as steady‐state attractors of regulatory systems dynamics. (a) Network of regulatory interactions for the Arabidopsis root stem cell niche as modeled in the García‐Gómez *et al*. (2020) paper (left). The network encompasses regulatory interactions between transcription factors as well as hormone (auxin and cytokinin) signals. Through starting from a large number of different initial conditions (different combinations of genes being expressed or not and hormone levels being high or not) and simulating the network dynamics (changes in gene and hormone levels) until they no longer change (steady state is reached), one finds the different network attractors or cell types as combinations of genes and hormones being expressed. This can be depicted either in a table (right, top) or on the network itself (right, bottom). (b) To understand where which cell type occurs and how the cell types of new cells that arise after division are dependent on position, the model regulatory network is embedded in a root topology. Each cell contains the same network of interactions, but different cells experience different superimposed auxin conditions (left). Additionally, SHORT ROOT (SHR) expression is limited to the vasculature. Together, these two conditions enable the cells to converge to the correct cell types. Furthermore, the model demonstrates that after division of the quiescent center (QC), one of the daughter cells can attain a different fate because of its different local neighborhood and that the fate attained depends on the division orientation and hence in which particular local context the new daughter cell is put (SHR at the top, auxin at the bottom).

For plant development, the work by the Alvarez‐Buylla group has been highly influential. Initially focusing on Arabidopsis flower development, network models were developed that incorporated all known regulatory interactions between flower development genes. These models were subsequently initialized from a large number of possible initial conditions (different combinations of genes being expressed or not) to identify the different attractors the model dynamics converge on in the long term. The models initially failed to find the complete set of attractors with gene expression patterns corresponding to sepal, petal, carpel, and stamen cell types. Therefore, the authors searched for those additional interactions and players that enabled the model to reconstitute the required flower organ attractors. In this manner, the models predicted missing interactions and directed future experimental efforts, as well as helped explain the temporal sequence and spatial patterns in which these cell types develop (Mendoza & Alvarez‐Buylla, 1998; Espinosa‐Soto *et al*., 2004; Álvarez‐Buylla *et al*., 2008; Díaz & Álvarez‐Buylla, 2021). For the plant root, the authors expanded their networks beyond transcription factors to also incorporate auxin and cytokinin (García‐Gómez *et al*., 2017) (Fig. 4a). The authors demonstrated that to reproduce QC, endodermis, peripheral and central vascular, and root cap cell types, known interactions were again insufficient and additional interactions were predicted. In a follow‐up study, an extended version of the regulatory model was applied to an idealized spatial root tip geometry to investigate asymmetric QC stem cell divisions (Fig. 4b). The spatial embedding of the model enabled the authors to demonstrate how a periclinal QC division orientation gives rise to a bottom daughter cell no longer receiving SHORT ROOT (SHR) influx from the vasculature, while in the case of an anticlinal division, both daughter cells keep having this influx. This difference in SHR influx explains the convergence of the daughter cells to different model attractors and thus cell types, and thus, why periclinal QC divisions are asymmetric whereas anticlinal ones are not (García‐Gómez *et al*., 2020).

#### Network motifs for biological computing and decision‐making

**Fig. 5:**
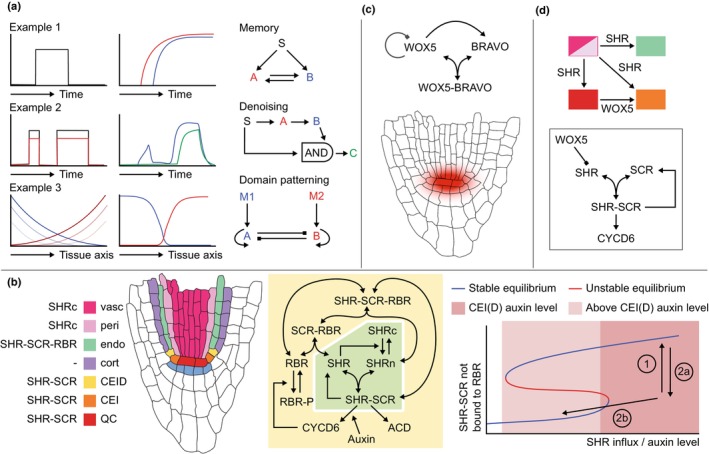
Network motif‐based decision‐making in cell type patterning and division orientation. (a) Network motifs relevant for decision‐making in developmental contexts. Example 1: if a transient signal induces two genes that mutually promote each other's expression, the transient signal can lead to a persistent response. Positive feedback thus leads to stable memory formation. Example 2: if an important gene C is only activated by a signal if both the signal is present and other intermediate genes A and B downstream of the signal are present (AND type integration of input), a precocious response to a brief noisy signal is prevented and C will only respond to a robust, longer lasting signal. Example 3: if two opposing morphogen gradients, M1 and M2, each induce genes A and B that both autoactivate, a decline in the morphogens would lead to the expansion of the A and B expression domains. This is prevented by the mutual repression between genes A and B enabling them to maintain distinct expression domains. Matching colors are used to link temporal dynamics and position in the regulatory motif of the different players. Fading colors in the example 3 indicate temporal decay of morphogen gradients. (b) Cruz‐Ramírez *et al*. (2012) aimed to explain the localized nature of asymmetric cell divisions in the cortex endodermis initials (CEI). The model network contains two positive feedback loops. The first feedback loop between SHORT ROOT (SHR) and SCARECROW (SCR) (green region in the network) results in a high nuclear SHR and SCR level in the endodermis that receives SHR influx, and low levels in the cortex where only very limited SHR arrives. The second feedback loop between SHR–SCR complex, auxin‐dependent CYCD6, and RBR (yellow region in the network) results in high nuclear SHR–SCR complex and CYCD6 levels in the CEI that experience high auxin, yet results in high SHR–SCR–RBR complex and low CYCD6 levels in the other endodermal cells that experience lower auxin due to their shootward position. During division, protein degradation is elevated in part of the cell cycle, causing SHR–SCR complex levels to decline (right figure, arrow 1). In the lower daughter cells with enduring high auxin, these levels restore after division (arrow 2a), whereas in the higher daughter cell that now experiences lower auxin levels, low levels are maintained (arrow 2b) and instead high SHR–SCR–RBR levels are established. (c) The models by the Ibanes laboratory (Betegón‐Putze *et al*., 2021; Mercadal *et al*., 2022) show how positive interaction between WOX5 and BRAVO can lead to stably maintained WOX5 and BRAVO expression near the quiescent center (QC) and stem cell niche while preventing it from spreading further, despite the mobile nature of WOX5. Essential is the complex formation between WOX5 and BRAVO, with only non‐complexed WOX5 having a negative feedback on itself and a positive feedback on BRAVO. (d) The van den Berg *et al*. (2021) model investigates the impact of SHR and WOX5 movement on QC and CEI divisions, incorporating a negative impact of WOX5 on SHR expression. Root illustrations in (b) and (c) are modified from the Plant Illustration repository (2017).

In addition to studying network attractors, zooming in on network submodules has also greatly contributed to our understanding of the functioning of biological systems. Seminal work by the Alon group demonstrated how network motifs can perform biological computation (Alon, 2007). Examples include how positive feedback enables amplification of and persistent responses to transient signals (i.e. memory formation), coherent feedforward loops allow the filtering out of signal noise, and mutual inhibition leads to the formation of distinct domains of gene activity. These insights have become part of mainstream biology (Fig. 5a).

In an elegant study by Cruz‐Ramírez *et al*. (2012), this approach was followed to investigate the asymmetric cell divisions (ACDs) producing the cortical and endodermal cells (Fig. 5b). Similar to the García‐Gómez *et al*. (2020) model, these authors could safely use a static nongrowing root tip model, as this is sufficient to address which patterning processes ensure that these ACDs only occur in the cortical endodermal initials (CEI) and not higher up along the endodermis (Fig. 5b). To achieve this, the authors incorporated a network of interactions between the involved transcription factors and cell cycle regulators based on their own and previously generated data. The authors identified two positive feedback loops in this network. The first consists of SHR, which induces SCARECROW (SCR) gene expression and SCR in turn promotes the nuclear localization of SHR necessary to control its gene expression (Fig. 5b, middle, green area). A second loop is formed by SCR‐inducing CYCLIN D6 (CYCD6) expression, with the CYCD6‐mediated phosphorylation of RETINOBLASTOMA RELATED (RBR) preventing the association with SCR that inhibits SHR‐SCR induced gene expression (Fig. 5b, middle, yellow area). The authors demonstrated that the first loop, which depends on SHR influx from the vasculature, ensures that SHR induces SCR and gets confined to the nucleus, restricting combined high SHR and SCR levels to the endodermal tissue layer. Next, the authors demonstrated that for the second loop to ensure high SHR–SCR complex and high CYCD6 levels to only occur in CEIDs, and high SHR–SCR–RBR levels not allowing for CEIDs elsewhere, an additional dependence of CYCD6 levels on auxin was necessary. Finally, using a separate single‐cell model, the authors showed how cell division induced the degradation of the major regulators ensures that more shootward‐oriented daughter cells that are exposed to lower auxin levels after cell division can change from a high‐to‐low SHR‐SCR‐CYCD6 state and thereby losing their ACD potential (Fig. 5b, right).

Recently, Winter *et al*. (2024) challenged the involvement of positive‐feedback‐mediated bistability in controlling endodermal ACDs. While their inducible SHR system allows for unprecedented quantitative insights into nuclear SHR and SCR dynamics, there are several problems with their interpretation of the Cruz‐Ramirez model. First, Winter *et al*. focus on detecting bistability in nuclear SHR and SCR levels to separate asymmetric from symmetrically dividing endodermal cells. However, the Cruz‐Ramirez model predicts this particular bistability to apply for endodermis vs cortex cells. Instead, differences between asymmetrically and normally dividing endodermal cells are predicted to relate to whether nuclear SCR is bound by RBR and thus prevented from inducing the high CYCD6 levels needed for ACDs. Secondly, the Winter *et al*. inducible SHR system results in ACD divisions along the entire meristematic endodermis, thus raising the question of whether bistability should even be expected.

Interestingly, later in development, middle cortex formation involves ACDs occurring along the entire endododermis, involving intermediate rather than high nuclear SHR levels (Koizumi *et al*., 2012). This suggests that the Winter *et al*. inducible SHR line can perhaps be interpreted as a precocious occurrence of middle cortex formation. For middle cortex formation, an alternative regulation of CYCD6 via PHABULOSA and GIBBERELLIC ACID (GA) has been proposed (Bertolotti *et al*., 2021). It would be interesting to see whether the incorporation of these players into the Cruz‐Ramirez model would be sufficient to simulate middle cortex formation and whether this indeed involves an alternative SHR state and different regulatory logic than for the CEI ACDs.

Still, the Cruz‐Ramirez paper predicts high SHR‐SCR and CYCD6 levels in both the CEI and the QC, while the QC *in planta* has no observable CYCD6 expression and is dividing at a much lower rate. Various other model studies have addressed the localized quiescent nature of the QC and how differences between the CEI and QC are generated. To investigate this, the Ibanes group (Betegón‐Putze *et al*., 2021; Mercadal *et al*., 2022) modeled the interplay between two quiescence‐conferring factors, WOX5 and BRASSINOSTEROIDS AT VASCULAR AND ORGANIZING CENTER (BRAVO). Using a single‐cell model, the authors demonstrated that the positive feedback between the two factors ensures their stable expression in the QC, with autorepression of both factors keeping absolute expression levels in check. However, given the (limited) mobility of WOX5, an important question is how WOX5 and BRAVO expression is kept from spreading beyond the QC and stem cell niche. In a follow‐up paper incorporating the gene regulatory network model into a spatial root tip layout, the authors demonstrated that key for this spatial confinement is the complex formation between WOX5 and BRAVO, which, due to the immobility of the complex, prevents further WOX5 movement and hence further activation of BRAVO and WOX5 (Fig. 5c). Note how this mechanism is similar to the SCR binding‐mediated immobilization of SHR I discussed earlier. Work from the Sozzani laboratory investigated the spatial and temporal control of CEI ACD and QC divisions (Van den Broeck *et al*., 2021). The developed model discerns vascular, QC, CEI, and endodermal cells and incorporates the transport of SHR from vasculature to QC, CEI, and endodermis cells, and the transport of WOX5 from QC to CEI cells (Fig. 5d). Results indicate that the repression of SHR by the mobile WOX5 is key to constraining CYCD6 expression and thereby ACDs. Interestingly, the model predicted that if QC divisions result in transient reductions in WOX5 levels, QC division rates may impact CEI WOX5 dynamics and thereby CEI ACD rates. In a similar vein, the model predicts that in a wox5 mutant, the more rapid restoration of SHR levels after divisions leads to increased CEI division rates. This latter prediction is in agreement with experimental data.

### 4. Root system branching

In addition to studying main root patterning, numerous modeling studies have investigated lateral root formation. Arguably, the most enigmatic stage in lateral root formation is its first step, the priming (pre‐patterning) of spatially semi‐regular sites competent for future lateral root formation through periodic elevations in auxin (signaling) levels. Experimental data suggest that the priming signal starts in the vasculature and is passed on to the pericycle from where lateral root development starts (De Smet *et al*., 2007). Experimental studies identified a major role for auxin precursor synthesis in the lateral root cap (Xuan *et al*., 2015), a strong correlation with lateral root cap apoptosis (Xuan *et al*., 2016), as well as an important role for auxin transport (De Smet *et al*., 2007). Together, this suggests auxin levels may need to vary and these variations may be related to growth and transport. However, in another early study, it was demonstrated that auxin (signaling) variations coincide with in‐phase and out‐of‐phase changes in a large number of genes (Moreno‐Risueno *et al*., 2010).

#### Repetitive patterning models

Repetitive pattern formation is among the most frequently modeled developmental patterns. In 1952, Alan Turing proposed his famous paper on ‘The chemical basis of morphogenesis’. While he laid out various mechanisms for repetitive pattern formation, arguably the most famous mechanism he proposed is for stable spot and stripe patterns arising from the interplay between a locally constrained activator and a more rapidly diffusing inhibitor. The mechanism predicts an inherent wavelength depending on activities and diffusivities of activator and inhibitor and hence predicts the periodic formation of a new spot or stripe in a polarly growing tissue each time a wavelength's worth of tissue has been added. While Turing was inspired by the regular patterning of shoot phyllotaxis, later research indicated that a positive feedback on PIN‐mediated feedback toward auxin maxima instead underlies phyllotaxis (see, e.g. Jönsson *et al*., 2006; Smith *et al*., 2006). While fundamentally different from a mathematical perspective, this can be interpreted as a special case of a substrate depletion type Turing patterning, where the buildup of activator in one location depletes it from other locations, thereby indirectly acting as its own inhibitor at a distance (Gierer & Meinhardt, 1972). Still, on the smaller spatial scales of epidermal pavement cell shapes and xylem cell wall patterning, or of epidermal trichome patterning, Turing mechanisms have been identified (Siero & Deinum, 2025).

**Fig. 6:**
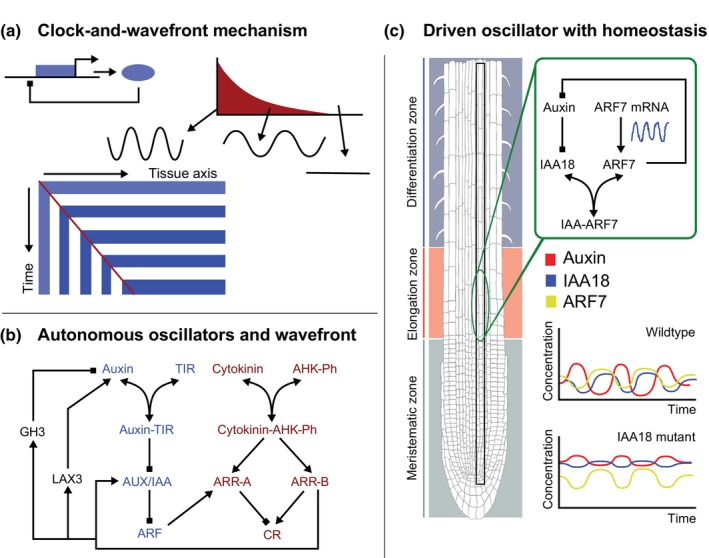
Clock‐and‐wavefront mechanism and related models for lateral root priming. (a) Illustration of a modern version of the clock‐and‐wavefront mechanism in which the cell‐autonomous clock is formed through negative feedback gene expression producing oscillations and the wavefront functions in slowing down and terminating oscillations, with cells memorizing the phase of the oscillation before termination. As the tissue grows at the tip, older cells are pushed out of the region of high morphogen gradient levels slowing down and subsequently stopping their oscillations, thereby transforming a temporally variable into a spatially variable pattern (space–time plot). (b) Compilation of the various oscillatory circuits proposed to underlie a root cell‐autonomous clock responsible for lateral root priming. In blue is the core network proposed by Middleton *et al*. (2010), in red the additions proposed by Muraro *et al*. (2011, 2013) to constrain oscillations to the non‐cytokinin (CK) signaling domain and in black are the alternative additions proposed by Mellor *et al*. (2016) to generate oscillations that do not critically depend on the upregulation of AUXIN/INDOLE‐3‐ACETIC ACID (AUX/IAA) by AUXIN RESPONSE FACTOR (ARF) and more critically rely on oscillations in auxin levels *per se*. (c) Schematic depiction of the driven root clock model developed in Perianez‐*Rodrigu*ez *et al*. (2021). The authors model a single vascular cell file with downward‐oriented PIN transporters, and within each cell a regulatory circuit encompassing auxin, IAA18, ARF7 mRNA, ARF7 protein, and IAA‐ARF interaction. The authors superimposed oscillatory dynamics for ARF7 mRNA levels specifically in the oscillation zone, having only a constant baseline mRNA production in the other regions, thus externally driving oscillations in a localized manner. For iaa18 mutants, due to failing auxin‐mediated IAA18 degradation, IAA18 levels hardly fluctuate while ARF levels are reduced and auxin levels are significantly elevated. Root illustration is modified from the Plant Illustration repository (2017).

Another classic mechanism for periodic pattern formation in growing tissue was proposed by Cooke and Zeeman to explain vertebrate somitogenesis (Cooke & Zeeman, 1976). In this clock‐and‐wavefront mechanism, each cell contains a cell autonomous clock or oscillator, while there is a tissue level wavefront affecting oscillator frequency. As cells, through growth, are pushed out of the morphogen maximum, oscillations cease, converting temporal oscillations into periodic patterns (Fig. 6a). While precise details remain debated (see, e.g. Cotterell *et al*., 2015), genes involved in the delayed negative feedback giving rise to gene expression oscillations as well as genes forming the wavefront have been identified (Palmeirim *et al*., 1997; Naiche *et al*., 2011).

Inspired by the observed gene expression oscillations, it was hypothesized that lateral root priming could also be driven by a cell autonomous clock (Moreno‐Risueno *et al*., 2010). To investigate this hypothesis, Middleton *et al*. (2010) developed a single‐cell model for canonical auxin signaling. Specifically, the authors incorporated the auxin‐mediated degradation of AUXIN/INDOLE‐3‐ACETIC ACID (AUX/IAA), and the resulting depression of AUXIN RESPONSE FACTOR (ARF)‐dependent gene expression, with AUX/IAA itself being one of the ARF upregulated genes (Fig. 6b, blue). The authors found that under sufficient auxin supply to prevent continuous high AUX/IAA and low free ARF levels, the model supports oscillations in free auxin, AUX/IAA, and ARF levels. Additional well‐known requirements for oscillatory dynamics are a sufficiently long time lag and nonlinearity in the regulatory circuit (Novák & Tyson, 2008). In an effort to combine the proposed clock with a wavefront, subsequent model extensions demonstrated how cytokinin antagonism could constrain auxin signaling oscillations to the high‐auxin low‐cytokinin domain of the root (Muraro *et al*., 2011, 2013) (Fig. 6b, burgundy). Importantly, these results are inconsistent with *in planta* data showing that the oscillation zone coincides with the early elongation zone where cytokinin signaling peaks. Thus, how cell‐autonomous oscillations in auxin signaling could be constrained to the oscillation zone remains an unanswered question. Another limitation of the proposed clock models is that oscillations require unrealistically high AUX/IAA transcription rates (Mellor *et al*., 2016). This requirement could be lifted by incorporating auxin signaling that induces GRETCHEN HAGEN 3 (GH3)‐mediated auxin degradation and LIKE AUXIN1 3 (LAX3)‐mediated auxin import (Mellor *et al*., 2016) (Fig. 6b, black). Instead, now clock oscillations require a slower induction of GH3 than of LAX3 to generate the necessary delays in the negative feedback loop. Since the model was developed in a single‐cell setting, it leaves open the question of how this mechanism may result in the required periodic auxin accumulation in early elongation zone vasculature cells where, due to rapid PIN mediated downward transport, localized auxin accumulations are expected to quickly dissipate (Deinum *et al*., 2016; Santos Teixeira *et al*., 2022). Indeed, in the only tissue level model simulating oscillations specifically in the early elongation zone, Perianez‐Rodriguez *et al*. (2021) combined a negative feedback auxin signaling network with superimposed elongation zone oscillations in ARF7 (Fig. 6c). By necessity, this disables the model from answering how oscillations are generated. However, the model does demonstrate that negative feedback may instead play a key role in auxin signalling homeostasis, keeping levels sufficiently low for priming to be discerned from background signalling levels.

**Fig. 7:**
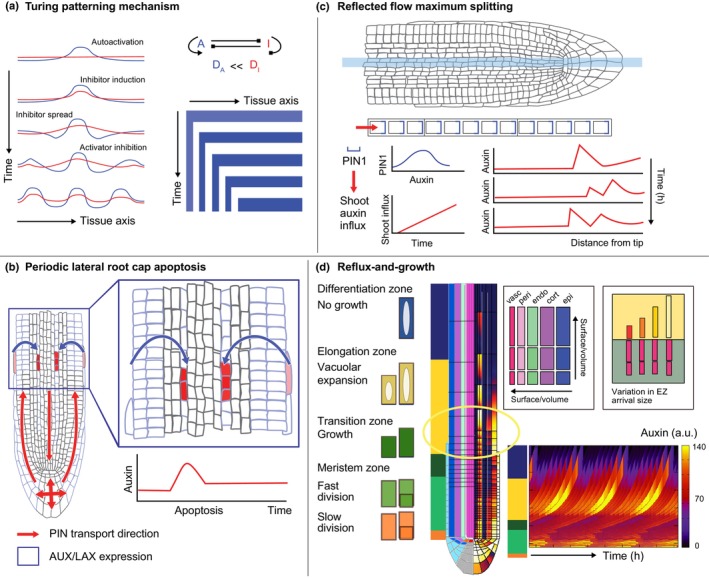
Turing patterning mechanism and related tissue‐scale lateral root priming mechanisms. (a) Illustration of a classical Turing patterning mechanism, with an activator (A, blue) that promotes its own levels and an inhibitor (I, red) that inhibits its own levels. In this particular example, the activator activates the inhibitor and the inhibitor inhibits the activator (network), causing their in‐phase patterning (line graphs). Small local elevations in activator level become amplified through autoactivation and result in the induction of the inhibitor. Due to its larger diffusion speed, the inhibitor is spread out to regions where activator levels are not high and the inhibitor effect exceeds the autoactivation effect resulting in activator depletion. As new elevations in activator arise, more activator peaks are formed until the tissue is filled with a regular ‘stripe’ pattern. In a tip‐growing tissue, the Turing mechanism results in the periodic production of new ‘stripes’ (space–time plot). (b) The so‐called reflected flow mechanism proposed by Mironova *et al*. (2010), in which PIN1 levels increase through auxin‐upregulated gene expression and decrease through auxin‐upregulated protein degradation, results in the formation of an auxin maximum at a distance from the tip. Combined with shoot‐derived auxin influx increasing over time, this results in the repeated splitting up of this maximum to produce new maxima. (c) Illustration of the periodic root cap apoptosis mechanism for lateral root priming proposed by Xuan *et al*. (2016), where apoptosing uppermost lateral root cap cells is supposed to excrete their internal auxin supplies to neighboring pericycle cells just before their apoptosis, which subsequently are transported to the vasculature. (d) In the reflux‐and‐growth mechanism, periodic elevations in early elongation zone (yellow zone) vascular cell file (dark pink cell file) auxin levels arise as an emergent property of tissue growth and auxin transport. The auxin reflux loop results in an auxin‐loading domain in the elongation zone (yellow oval). Cell expansion in this elongation zone causes an increase in the cell surface‐to‐volume ratio and hence passive auxin uptake capacity, with this increase being the largest in narrow vasculature cells. Root tip growth dynamics result in periodic variations in the sizes at which cells enter the elongation zone. Combined, this results in periodic increases in vascular auxin uptake capacity. Root illustrations in (b) and (c) modified are from the Plant Illustration repository (2017).

Inspired by Turing patterns (Fig. 7a) and models for phyllotaxis, other lateral root modeling studies have instead focused on the potential role of auxin transport and/or tissue growth dynamics in generating periodic variations in auxin levels as an emergent property rather than from cell autonomous oscillations. Mironova *et al*. (2010) built a 1D model of the root vasculature incorporating cell growth and division as well as downward PIN1‐mediated auxin transport. Experimental data indicate that both PIN transcription and PIN protein degradation are promoted by auxin, with the latter requiring higher auxin levels than the former. Incorporating this optimal PIN1 auxin dependence in the model, it was found that as downward auxin transport causes bottom auxin levels to accumulate, PIN1 levels locally decline, and an auxin maximum forms in a self‐organized manner at a distance from the tissue boundary. Next, the authors assumed that plant growth results in a continuous increase in the auxin supply the root receives from the shoot and incorporated this into their model. This resulted in a destabilization of the auxin and PIN1 pattern, with the expansion and splitting up of the auxin maximum leading to the periodic generation of new auxin maxima (Fig. 7b). While *in planta* for the QC auxin maximum no spatial shifting and splitting up have been observed, possibly the model results may instead apply to oscillation zone auxin dynamics. In a study by Xuan *et al*. (2016), a root scale model of auxin dynamics was developed to investigate the potential impact of periodic lateral root cap apoptosis on vascular auxin dynamics (Fig. 7c). When assuming that all auxin of the uppermost root cap cells is extruded to the neighboring epidermis cell before its apoptosis, the model displayed a modest increase (maximum 7%) in vascular auxin levels. Still, the fact that experimentally SOMBRERO (SMB) mutants display only slightly less regular priming sheds doubt on the causal nature of apoptosis for priming.

Finally, van den Berg *et al*. (2021) developed a root tip model combining the details of cellular auxin import and export dynamics with cell growth, division, and elongation dynamics (Fig. 7d). Relative to earlier models from our group, root growth dynamics were modeled in more detail, distinguishing within the meristem between slow‐dividing initial cells, rapidly dividing transit amplifying cells, and growing but no longer rapidly dividing transition cells. In this model, periodic elevations in vasculature auxin levels arose as an emergent property. Through reducing PINs in outer tissue layers, we could show that these oscillations required a functional reflux loop with upward‐ and inward‐mediated transport at the end of the meristem resulting in auxin loading in the early elongation zone. An advantage of our non‐mechanically realistic growth model was that it enabled us to block growth in some cell files but not others, something clearly impossible *in planta* or in a mechanically more realistic model. This allowed us to demonstrate that the growth and elongation of vascular cells but not other cells is crucial for oscillations. To investigate the mechanism underlying the periodic nature of the auxin elevations, we studied cell size dynamics in our model. We observed variations in the sizes with which cells arrive in the early elongation zone that for vascular cells correlated strongly with the auxin levels attained by these cells. These cell size variations naturally arise from differences in timing relative to their latest round of division and are also observed experimentally (Goh *et al*., 2023). We hypothesized that these cell size differences result in differences in membrane area and hence passive auxin uptake capacity, and that this explains why narrow vascular cells which undergo the most dramatic surface‐to‐volume ratio increase show the strongest increase in auxin levels. Supporting this idea, artificially keeping passive auxin uptake constant despite increases in membrane area or widening vascular cell width and hence reducing surface‐to‐volume ratio were found to substantially reduce oscillation amplitude. In an extension of the model (Santos Teixeira *et al*., 2022), model cells were endowed with a hypothetical epigenetic factor enabling them to temporally integrate their experienced auxin levels, with high levels of this factor subsequently upregulating auxin signaling capacity and downstream gene activation. These additional interactions enabled stable memorization of priming despite cells through growth being displaced to an ever lower auxin context. The hypothesized memorization process may fit well with recent findings that prebranch sites (PBS) depending on light conditions may lose as well as regain auxin signaling competence (Ren *et al*., 2024). Additionally, the proposed combined mechanisms offer a reconciliation between the conflicting views on whether auxin levels or rather auxin‐related gene expression oscillates, suggesting that it starts with the first and is memorized by the latter. While model predictions with regard to priming frequency and spacing have been experimentally validated, so far it has been technically unfeasible to measure vascular cell size and auxin loading dynamics.

## III. Discussion

In this review, I have discussed various models that have contributed to our understanding of plant root development. Additionally, I explained how these are grounded in classical developmental and theoretical biology concepts such as morphogen gradients, cell types as attractors, decision‐making motifs, oscillators, and Turing patterns yet also sometimes had to push beyond these classics to explain the full details of the plant system. I attempted to demystify model construction by explaining the rationale and approach behind choosing what does or does not go into a mechanistic model and how this is further aided through iterating between models and experiments. I apologize to all those authors of great modeling work that I could not discuss owing to space limitations.

Classically, mechanistic models aiming to understand plant developmental processes have been hypothesis‐driven. Their development starts from certain knowledge of the key players and interactions involved in the process one aims to understand. The models then serve to investigate whether these are necessary and sufficient, and if not, what type of players or interactions could be missing. Finally, these models are a powerful tool to reveal unexpected emergent properties. Typically, to maintain tractability and computational efficiency as well as keeping the number of parameters within bounds, modelers strive to limit the number of variables these models contain.

By contrast, over the last decades, biology has become an extremely data‐rich science, with exponentially accumulating volumes of data on genome organization, gene expression, translation, phosphoproteomics, chromatin organization, TF binding, etc. Thus, as an alternative to a hypothesis‐driven approach, unbiased data‐driven approaches are gaining more and more importance. For developmental biology, analysis of single‐cell gene expression dynamics using dimension reduction techniques to identify cell types (van der Maaten & Hinton, 2008; Becht *et al*., 2019) and using pseudotime to reveal developmental trajectories of cell fate acquisition (see, e.g. Trapnell *et al*., 2014) has proven extremely powerful. These approaches are now becoming even more revealing with the inclusion of the spatial dimension in the data acquisition process (Ståhl *et al*., 2016; Tian *et al*., 2023). Although producing valuable insights, in isolation these analyses remain largely descriptive.

Moving toward mechanism, gene expression data are frequently used to derive the architecture of the underlying regulatory network, revealing which genes are regulating each other. With the recent advent of ever more sophisticated machine learning (ML) techniques, even differential equations may be learned from gene expression dynamics (Brunton *et al*., 2016; Hossain *et al*., 2024), providing insights into the mechanisms controlling the dynamics of these regulatory interactions. However, generating a sufficiently large number and density of time points for gene expression analysis to allow this type of network and equation inference is far from trivial in many systems. Additionally, due to the many levels of regulation besides transcription not captured by these algorithms, data‐derived network architectures and equations may not necessarily correspond to the actual mechanisms of regulation.

Thus, rather than data‐driven approaches replacing hypothesis‐driven mechanistic modeling, great promise lies in their combination, combining the strengths of both methods and thereby overcoming their respective weaknesses (Baker *et al*., 2018; Alber *et al*., 2019). While thus far hybrid mechanistic‐machine learning models have been mostly applied outside of the field of plant developmental biology, I expect these integrated approaches to gain substantial traction in the coming years and to enable us to further push the boundaries of plant developmental modeling.

## Competing interests

None declared.

## Author contributions

KHT conceived and wrote the manuscript.

## Disclaimer

The New Phytologist Foundation remains neutral with regard to jurisdictional claims in maps and in any institutional affiliations.
